# Supplementary material for: Risk factors for emerging intraocular inflammation after intravitreal brolucizumab injection for age-related macular degeneration
Source: PLoS One. 2021 Dec 6;16(12):e0259879. doi: 10.1371/journal.pone.0259879 (PMC8648104; doi:10.1371/journal.pone.0259879)
Supplement: S1 Table — (PDF) [file pone.0259879.s001.pdf]

	Total	Treatment Naïve	Switch
Patients	90	72	18
Eyes	93	75	18
Age	74.8±9.0	75.0±8.8	72.1±8.8
Female/Male	15/75	11/61	4/14
Lesion Type			
Typical	32	22	10
PNV	14	11	3
PCV	47	42	5
Previous treatment			
Aflibercept (eyes)			18
Averaged number of injection			28.7 ±19.3
PDT or RF-PDT (eyes)			5
Averaged number of (RF)PDT			3.2±3.3
