# Supplementary material for: Risk factors for emerging intraocular inflammation after intravitreal brolucizumab injection for age-related macular degeneration
Source: PLoS One. 2021 Dec 6;16(12):e0259879. doi: 10.1371/journal.pone.0259879 (PMC8648104; doi:10.1371/journal.pone.0259879)
Supplement: Attachment — Submitted filename: @Dear_Dr.docx [file pone.0259879.s002.docx]

1. Please ensure that your manuscript meets PLOS ONE's style requirements, including those for file naming. The PLOS ONE style templates can be found at

<https://journals.plos.org/plosone/s/file?id=wjVg/PLOSOne_formatting_sample_main_body.pdf> and

https://journals.plos.org/plosone/s/file?id=ba62/PLOSOne_formatting_sample_title_authors_affiliations.pdf

We affirm that all the formatting changes required by PLOS ONE have been incorporated in our revised manuscript.

2. Thank you for including your ethics statement: "This retrospective study obtained institutional review board approval from the Gunma University Graduate School of Medicine.The data were analyzed anonymously"

a) Please provide additional details regarding participant consent. In the ethics statement in the Methods and online submission information, please ensure that you have specified (1) whether consent was informed and (2) what type you obtained (for instance, written or verbal, and if verbal, how it was documented and witnessed). If your study included minors, state whether you obtained consent from parents or guardians. If the need for consent was waived by the ethics committee, please include this information.

If you are reporting a retrospective study of medical records or archived samples, please ensure that you have discussed whether all data were fully anonymized before you accessed them and/or whether the IRB or ethics committee waived the requirement for informed consent. If patients provided informed written consent to have data from their medical records used in research, please include this information.

Once you have amended this/these statement(s) in the Methods section of the manuscript, please add the same text to the “Ethics Statement” field of the submission form (via “Edit Submission”).

For additional information about PLOS ONE ethical requirements for human subjects research, please refer to <http://journals.plos.org/plosone/s/submission-guidelines#loc-human-subjects-research>.

 We have added the necessary statement in Methods (Lines 59–60).

“The requirement for written informed consent was waived owing to the retrospective nature of the study.”

[Note: HTML markup is below. Please do not edit.]

Reviewers' comments:

Reviewer's Responses to Questions

**Comments to the Author**

1. Is the manuscript technically sound, and do the data support the conclusions?

The manuscript must describe a technically sound piece of scientific research with data that supports the conclusions. Experiments must have been conducted rigorously, with appropriate controls, replication, and sample sizes. The conclusions must be drawn appropriately based on the data presented.

Reviewer #1: Partly

Reviewer #2: No

2. Has the statistical analysis been performed appropriately and rigorously?

Reviewer #1: I Don't Know

Reviewer #2: I Don't Know

3. Have the authors made all data underlying the findings in their manuscript fully available?

The [PLOS Data policy](http://www.plosone.org/static/policies.action#sharing) requires authors to make all data underlying the findings described in their manuscript fully available without restriction, with rare exception (please refer to the Data Availability Statement in the manuscript PDF file). The data should be provided as part of the manuscript or its supporting information, or deposited to a public repository. For example, in addition to summary statistics, the data points behind means, medians and variance measures should be available. If there are restrictions on publicly sharing data—e.g. participant privacy or use of data from a third party—those must be specified.

Reviewer #1: No

Reviewer #2: No

4. Is the manuscript presented in an intelligible fashion and written in standard English?

PLOS ONE does not copyedit accepted manuscripts, so the language in submitted articles must be clear, correct, and unambiguous. Any typographical or grammatical errors should be corrected at revision, so please note any specific errors here.

Reviewer #1: Yes

Reviewer #2: Yes

5. Review Comments to the Author

Please use the space provided to explain your answers to the questions above. You may also include additional comments for the author, including concerns about dual publication, research ethics, or publication ethics. (Please upload your review as an attachment if it exceeds 20,000 characters)

Dear Dr. Vavvas,

Thank you for giving me the opportunity to submit a revised draft of my manuscript titled “**Risk factors for emerging intraocular inflammation after intravitreal brolucizumab injection for age-related macular degeneration**” to *PLOS ONE.* We appreciate the time and effort that you and the reviewers have dedicated to providing your valuable feedback on my manuscript. We are grateful to the reviewers for their insightful comments on our paper. We have been able to incorporate changes to reflect most of the suggestions provided by the reviewers. We have indicated the changes within the manuscript in red font. Here is a point-by-point response to the reviewers’ comments and concerns.

Reviewer #1: The authors present an interesting study on a topic of very high interest: intraocular inflammation after brolucizumab. They are invited to consider the following recommendations to further strengthen their work:

1. The very title of the article is not in agreement with the article. Do the authors investigate bevacizumab (Avastin) or Brolucizumab (Beovu) ?

We sincerely apologize for the error in the title and have rectified the mistake in typing. We have used Brolucizumab in our study and have analyzed the IOI after brolucizumab injection.

2. Define ’others’ in abstract results

We agree that the term “other” was vague, and we have replaced it with the term “non-IOI group” on Line 29 of our manuscript.

3. Please correct syntax in the last phrase of your abstract: ‘elders’ is not a ‘risk factor’.

We replaced the term “elders” with “old age” to describe the risk factor on line 37 of the abstract.

4. Please be more specific in your introduction tha, ‘relatively frequently’.
Describe the percentages for IOI for bevacizumab, ranibizumab and aflibercept and directly compare with the percentages in the brolucizumab studies.

Thank you for your advice. We have added percentages of incidence of IOI after brolucizumab, ranibizumab and aflibercept injections, as follows (Lines 50–53):

“However, in these reports, unexpected cases of intraocular inflammation (IOI) after intravitreal brolucizumab (IVBr) injection have been noted having an incidence of 4.6% [12]; this was higher than that of ranibizumab and aflibercept injection groups (1.5% and 0.5-1.1 % respectively) [13].”

5.Seems like not all the eyes included in this study were treatment naive. if not, how many weeks/months since the last antiVEGF injection? define your inclusion and exclusion criteria in more detail.

Eighteen non-treatment naïve eyes (19%) were included in this study. In 15 eyes, prior to introducing brolucizumab, at least 3 monthly injections of aflibercept were performed, however the eyes did not respond to the treatment. The average number of injections was 28.7 ± 19.3 injections in these eyes. In 5 eyes, PDT or RD-PDT with aflibercept was performed (3.2±3.3). We have added these results in Supplementary Table 1.

6. Was FA, IA and SS-OCTA performed to all eyes at every single visit ? This would be interesting in terms of how many imaging tests were the patients undergoing in their regular visits, since this was a retrospective review, not a prospective study.

We added the missing information in the Methods (Lines 80–85).

“IOI includes iritis, vitritis, and vasculitis or occlusion of vessels. To precisely detect IOI, a detailed slit lamp test and fundus examination were performed. Additionally, we analyzed the patients’ eyes with an ultra-wide field scanning laser ophthalmoscope (Optos 200Tx; Optos, Dunfermline, Scotland) in detail, during monitoring, to broadly detect vascular changes. To diagnose occlusion of vessels as IOI, we performed FA and IA angiography at the initial visit and at the visit after the loading dose. SS-OCT were performed at every visit.”

7. Methods should be overall more detailed.
What exactly was the technique/protocol followed when injecting?
Were all the injections preformed by the same person?
If not, what was the technique/protocol of all different treating physicians exactly the same ?

As per your comments, we have incorporated the following information in the revised manuscript.

Lines 71–76: In In treatment naïve cases, after three months injections, patients were followed-up using Treat and Extend (TAE) regimen. If persistent signs of active disease were present, injections were continued bimonthly until the macula was dry. Once the macula was dry, the interval between injections increased to 4 weeks initially and the maximum interval was 16 weeks. Contrastingly, for non-treatment naïve cases, TAE regimen was applied after first brolucizumab injection.

Lines 77–78: “In this study, 7 physicians performed IVBr, in accordance with guidelines for intravitreal injection for macular disease.”

In addition, we have previously published a paper^#^ which aimed to standardize intravitreal injections and all staff were trained using the manual prior to performing intravitreal injections.

#. Ganka Rinsho Kiyo　2018 Vol 11,No 9 P.688 (Domestic journal)

8. You should define intraocular inflammation in a very strict and precise manner
Thank you for the advice. We have included the definition of IOI in the revised manuscript.

Lines: 80–85: “For the purpose of our study, IOI included iritis, vitritis, and vasculitis or occlusion of vessels. To precisely detect IOI, a detailed slit lamp test and fundus examination were performed. Additionally, we analyzed the patients’ eyes with an ultra-wide field scanning laser ophthalmoscope (Optos 200Tx; Optos, Dunfermline, Scotland), during monitoring, to broadly detect vascular changes. To diagnose occlusion of vessels as IOI, we performed FA and IA angiography at the initial visit and at the visit after the loading dose. SS-OCT were performed at every visit.We added it in methods.”

9. It would be interesting to investigate whether the injection technique/protocol had any effect on the occurrence of IOI.
Thank you for this suggestion to investigate whether the injection technique/protocol had any effect on the occurrence of IOI. It would have been interesting to explore this aspect. However, in the case of our study, it seems slightly out of scope because, in our department, the method for intravitreal injections by every physician was precisely standardized, and we further believe that different protocols do not have an effect on the emergence of IOI.

But this particular topic can be discussed in detail in future work.

10. Statistical analysis: did the authors check whether their data are normally distributed ?

In IOI cases, the number of cases was limited, and the data did not show a normal distribution.

11. The authors need to go more in depth and try to explain why the factors they found that were associated with higher IOI rates are risk factors. Please elaborate more in your discussion.

Iritis, OCV, vasculitis, and occlusion of retinal vessels can be observed in IOI; also, inflammatory cells (B cells, T cells and macrophage) were detected in the vitreous in cases of IOI. Vasculitis and occlusion of retinal vessels seems to be deeply associated with the pathogenesis of diabetes. Chronic microinflammation with infiltration of microglia or macrophages is involved in the pathogenesis of diabetic retinopathy. In addition, iritis sometimes developed in patients with severe diabetes mellitus. Considering all these factors, diabetes mellitus can potentially promote inflammatory processes. Thus, we have modified the discussion as follows:

Lines 157–171: “Diabetes can accompany microangiopathy and macroangiopathy, which are caused by sclerosis [27] [28] . Hyperglycemia due to diabetes, tissue resistance against insulin, hyperinsulinemia accompanied with insulin tolerance, and obesity, specifically visceral fat obesity, can promote arteriosclerosis in diabetes [29]. Mortality due to coronary artery diseases and the frequency of cardiac infarction increase in cases of diabetes [30]. Advanced glycation end-products or glycation proteins can induce the release of pathophysiological substances such as IL-6, IL-8, MCP-1, ICAM-1, and IP-10 in conditions such as diabetic retinopathy [31]. In addition, microinflammation can cause diabetic retinopathy mediated by circulating monocytes, tissue-resident macrophages, and monocyte-derived inflammatory macrophages [32]. Patients with severe diabetes mellitus sometimes have accompanying iritis clinically, with hyperpermeability due to impairment of blood aqueous humor barrier due to damage of endothelial cells in capillary vessels. Histopathological sections of the vitreous, aspirated from patients with vasculitis that developed after brolucizumab injection, revealed infiltration of CD20 positive B cells, CD3, 4, and 8 positive T cells and CD68 positive histiocytes [33]. IOI consists of iritis, OCV, vasculitis, and vascular occlusion, and such pathogeneses if accompanied by diabetes mellitus can potentially promote further inflammation.”

Reviewer #2: The authors present a study regarding "Risk factors for emerging intraocular inflammation after intravitreal bevacizumab injection for age-related macular degeneration". This is a very interesting topic as we are trying to understand the safety profile of this new agent Brolicizumab even though they have the wrong name of medicine in the title which makes it very confusing...

However, the study the authors have presented has very limited analysis and does not add anything to the literature and is missing significant information eg time when IOI was diagnosed, severity of IOI, number of previous anti VEGF injection, what type of of anti VEGF injection, also majority of oateintas had PCV in the IOI group- was it the same as in the IOI group? What about degreee of existing DR in addition to DM. Authors should do a much more through review of all characteristics of patients to provide an analysis of the risk factors in involved in IOI group.

We sincerely apologize for the error in the title and have rectified the mistake in typing. We have used Brolucizumab in our study and have analyzed the IOI after brolucizumab injection.

As shown in Table 2, IOI was detected at 20.5±2.2 days after brolucizumab injection. In 7 eyes, IOI developed after 1^st^ injection and in 4 eyes at 2^nd^ injection and in 3 eyes at 3^rd^ injection.

The extent of inflammation was also described in Table 2; 8 eyes showed iritis, 11 eyes revealed vitritis, 10 eyes with arteritis, 6 eyes with periphlebitis and 3 eyes showed vascular occlusion.

In this study, 18 eyes were non-treatment naïve cases and we have added the information of previous treatment and lesion type in Supplemental Table1.

“Eighteen non-treatment naïve eyes (19%) were included in this study. In 15 eyes, prior to introducing brolucizumab, at least 3 monthly injections of aflibercept were performed, however the eyes did not respond to the treatment. The average number of injections were 28.7 ± 19.3 injections in these eyes. In 5 eyes, PDT or RD-PDT with aflibercept was performed (3.2±3.3).”

We have also added information on the severity of diabetic retinopathy in Table 2, and there seems to be no correlation in severity of diabetic retinopathy and IOI or HbA1c, because the cases with vascular occlusion neither had diabetic retinopathy nor high values of HbA1c.

We have analyzed lesion types between IOI or non-IOI, however, there was no correlation among them.　We have added this analysis to Table 1.

There has been substantial modification to the methods, discussion, and tables of the paper in line with the reviewers’ suggestions, and we hope that the paper will benefit from these revisions. Once again, we thank you for the time you put in reviewing our paper and look forward to meeting your expectations.
