# Supplementary material for: Risk factors for emerging intraocular inflammation after intravitreal brolucizumab injection for age-related macular degeneration
Source: PLoS One. 2021 Dec 6;16(12):e0259879. doi: 10.1371/journal.pone.0259879 (PMC8648104; doi:10.1371/journal.pone.0259879)
Supplement: Attachment — Submitted filename: @Reviewer.docx [file pone.0259879.s003.docx]

Reviewer #1: The authors have substantially improved their work. They are invited to consider the following additional recommendations:
1. Your abstract’s conclusion seems more like a purpose. Please provide a conclusion that sums up the findings of your study and why they might be clinically relevant. (same way as in the conclusion ion the manuscript)

**Response:**

In our cohort, three factors might have influenced the occurrence of IOI after IVBr injections. IOI with vascular occlusion by itself did not seem to cause severe visual impairment in this cohort. Thus, it is likely that these factors were not associated with vision threatening in the eyes with IOI. Considering the possibility of further indication of IVBr for diabetic macular edema, “Further studies are required to investigate potential risk factors for IOI” should be added to the conclusion. We have added this information to the Abstract and Discussion section, as follows

“The comparison of the clinical profiles of IOI or non-IOI cases in IVBr treatment for AMD suggests that the risk factors for IOI are old age, female sex, and history of diabetes; however, IOI with vasculitis or vascular occlusion in this cohort does not seem to cause severe visual impairment. Further studies are required to investigate the potential risk factors for IOI.”

2. Injection technique varies between different treating physicians. This is well documented for antiVEGF injections. How can the authors be sure that injection technique did not affect the rates of IOI since per line 72, 7 different physicians performed the brolucizumab injections.

**Response:**

As we responded to your comment in the previous revision, we published an article that aimed to standardize the injection techniques for individual physicians. All staff were trained using this manual. We performed 4093 injections of anti VEGF drugs, except for brolucizumab, for one year and no IOIs occurred. We also participated in a multicenter study, which investigated the incidence of endophthalmitis after anti VEGF injections in 2020, but no infections were reported during this period. These results suggested that standardization was successful.

Ganka Rinsho Kiyo　2018 Vol 11,No 9 P.688 (Domestic journal)

Sci Rep 2020 Dec 17;10 (1): 22122

3. Please clarify the device used for SS-OCT (line 80)

**Response:**

We used DRI-OCT Triton (Topcon, Tokyo, JAPAN). We have added this information to the Methods section.

4. Table 2 is confusing. What do all the ‘1’s represent ? If its individual parents please number the patients.
**Response**

We erased “1” and added “+” or “0” instead.

5. Did the number of previous injections in the non-treatment naive eyes affect the rate of IOI ?

**Response:**

In two patients with IOI in the non-treatment naïve eyes, one month prior to brolucizumab injection, aflibercept was injected. Just before brolucizumab injection, no IOI was detected on slit lamp and fundus examination and optical coherence tomography. IOI in these two cases occurred on 12- and 21-days post brolucizumab injections. Thus, we believe that IOI was mostly associated with brolucizumab injection.

Reviewer #2: Even though the manuscript has improved from pervious version, it still lacks in depth analysis in particular regarding the vision outcomes of IOI and the vision threatening forms of IOI of vasculitis and how they responded and if risk factors described for all IOI also apply for the vision threatening one that is the most concerning

**Response:**

Thank you so much for your comment. First, IOI with vascular occlusion in this cohort did not seem to cause severe visual impairment. Additionally, IOI with vasculitis did not worsen the visual acuity. Early treatment with steroids could relieve IOI and restore visual outcome.

In two cases with decreased vision after IOI, exudative change of MNV seemed to contribute to the visual deterioration. Finally, old age, female sex, and history of diabetes seemed to contribute to the occurrence of IOI in this cohort; however, these factors did not seem to be associated with vision threatening in the eyes with IOI.

We have added this description to the Discussion section.
